# Editorial and Miscellaneous

**Published:** 1864-06

**Authors:** 


### C. S. Medical & Surgical Journal.

RICHMOND, JUNE, 1864.

*AYRES & WADE .....PUBLISHERS AND PROPRIETORS.*

### *Conservative Surgery in Compound Fracture of Femur.*

The military surgeon has no question submitted to his discretion of more importance than to determine upon the propriety of amputation in compound fractures of the femur, the result of gun-shot wounds.

The authorities, both French and English, teach us not to trust these cases to nature, and broadly state that, in the operation alone, there is hope; but the statistics, particularly of the Crimean campaign and in our own service, prove that the mortality after amputation is enormous, and force us to consider the propriety of conservative practice in this numerous class of surgical accidents.

In various numbers of the Journal, the reader will find many interesting observations bearing upon this question, and we submit at this time a consolidated statement of compound fractures of femur treated without operation, compiled from the records in the Surgeon-General's office, from June, 1862, to Feb. 1, 1864, inclusive. We have, in this summary excluded all cases not positively determined, and hence, while the number of observations is greatly reduced, the value of the conclusion is increased in like proportions.

Total number of cases, 221—recovered, 116—52 per cent.

Average period of recovery, 104 days—greatest period, 255 days, and least, 41 days. Average period where death occurred, 52 days—greatest period, 185 days—least, one day. Average amount of shortening, 1 9-10ths inches—greatest, 5 inches, and least, half an inch.

When we compare directly the results of amputations with the table of cases not operated on, we feel still more disposed to rebel against the authority of Guthrie, McLeod, Larrey, Percy and Dupuytren, and at least hesitate before condemning the shattered limb to instant ablation. Our own statistics are as follows:

507 cases amputated—250 recovered—50 per cent.

221 cases *not* amputated—116 recovered—52 per cent.

The chance for life being more than equal, the value of the leg saved should be considered, and the table throws important light on this point—the average shortening is less than *two inches.*

Submit these facts to an intelligent soldier—"your thigh is broken by the ball—your chances of life are even whether amputation is performed or not—without the operation, if you live, you will suffer an average of 104 days—if you die, it will take 52 days—and when you recover, you will have a leg two inches shorter than the other." It is easy to imagine his reply to this simple statement of the facts—"Give me a chance for life *and* limb."

The very important remarks on this subject from the most recent French authority on military surgery, published in the chronicle for this number, corroborate forcibly the position which is assumed in this article. The reader's careful attention is called to this interesting translation, but for the sake of condensation, we groupe his statistics with those collected from various sources during the war.

*Legouest.*

1,664 cases amputated—recovered, 123—7 per cent.

337 cases not amputated—recovered 117—31 per cent.

*Chimborazo Hospital Statistics—first and second numbers of this Journal.*

31 cases not amputated—recovered, 19—61 per cent.

These observations bring us to conclude, that whenever, in compound fracture of femur, the result of gun-shot-wound, there is a doubt as to the propriety of amputation, that we give the leg the benefit of the doubt—the chances of life being at least equal, and the value of the limb, after recovery, being worth the effort to save it.

